# Hypercalciuria and Nephrocalcinosis as Early Feature of Wilson Disease Onset: Description of a Pediatric Case and Literature Review

**DOI:** 10.5812/hepatmon.6233

**Published:** 2012-08-25

**Authors:** Valeria Di Stefano, Elena Lionetti, Novella Rotolo, Mario La Rosa, Salvatore Leonardi

**Affiliations:** 1 Department of Pediatrics, University of Catania, Catania, Italy

**Keywords:** Hypercalciuria, Nephrocalcinosis, Hepatolenticular Degeneration, Polyuria, Osteoporosis

## Abstract

**Background:**

Wilson’s disease (WD) is a rare autosomal-recessive disorder characterized by a mutation in the ATP7B gene, located on chromosome 13, which encodes a protein involved in the metabolism of copper.

**Case Presentation:**

We described the case of an Indian male with a history of polydipsia and polyuria, related to hypercalciuria and consequent nephrocalcinosis. The symptoms began at the age of five years old, but he was not diagnosed with WD until he reached an adolescent age. We started therapy with D-Penicillamine, B-vitamin complex and recommended a low copper diet. Renal involvement in Wilson’s disease, characterizing by hypercalciuria, was firstly reported by Litin in 1959.

**Conclusion:**

Our case was different and peculiar from the previously described cases because the patient presented a very long history (10 years) of permanent hypercalciuria without any acute episode of nephrolithiasis.

## 1. Background

Copper is an essential element for cellular function, to the extent that being free of copper is extremely toxic and can produce irreversible cellular damage on various organs and tissues and may produce a strikingly diverse clinical picture. Molecular genetic analysis reveals at least 300 distinct mutations for the Wilson’s disease (WD). Many affected individuals are actually compound heterozygotes, having inherited different mutation from each parent. It is still unclear whether the different mutations are responsible for the variability of clinical studies or the age of the patient, when symptoms of WD appear. The disease rarely begins before five years old and its appearance depends on the accumulation of free copper in the organs (1, 2). In this study, we described the case of an Indian male with a history of polydipsia and polyuria, related to hypercalciuria and consequent nephrocalcinosis, which began at the age of five years old but unfortunately was diagnosed in adolescent.

## 2. Case Presentation

**Figure 1:**
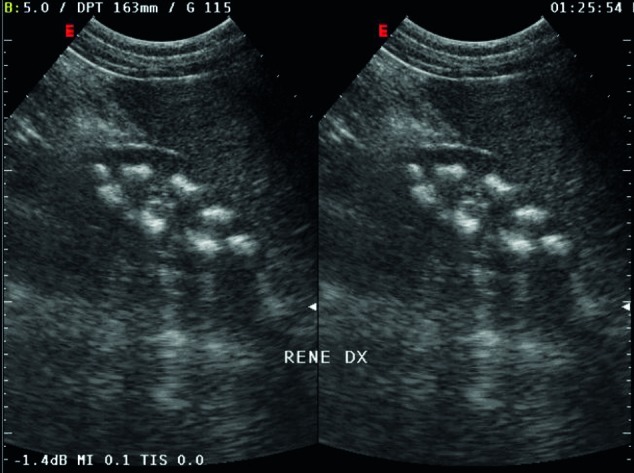
Kidney Ultrasonography Showing the Presence of Bilateral Nephrocalcinosis

**Figure 2:**
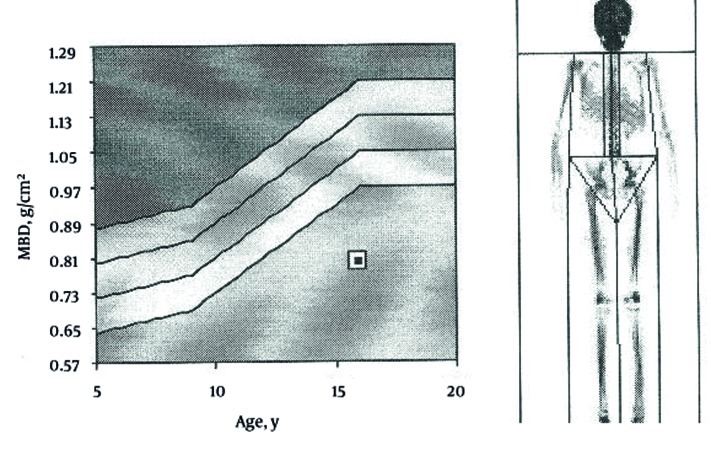
Bone Density Scan Revealing Significant Bone Demineralization, Z-SCORE -4.1

A 16 years old boy came to our attention because of polyuria (about eight liters of urine) and polydipsia associated with asthenia. There was family history of diabetes mellitus for the father and allergy to inhalants from the mother. He was second-born by normal delivery of consanguineous parents (first degree cousins); weight at birth was 3015 gr. From the age of five, the symptoms of polydipsia and polyuria have increased. At 10 years old, he suffered from a fracture of his left foot after a trauma of mild intensity. A physical examination revealed evidence of growth cessation. The patient’s weight and height were below the third percentile of growth (35, 4 Kg; 143 cm) and delayed puberty was diagnosed (absent pubarche, testicular volume 6-8 cc, penis stage was the third). At the time of admission, the patient’s clinical condition was poor, skin turgor was reduced, muscles appeared norm tonic but slightly hypertrophic. Thoracic, cardiac and abdominal examinations were normal. There was no clinical sign of neurologic diseases. Speech ability and school performance were good. Laboratory findings at admission were normal; the only abnormal values were hypophosphatemia, hypokalemia and hyper transaminasemia: (Aspartate Aminotransferase (AST) 47 U/L and Alanine Aminotransferase (ALT) 81 U/L while the normal ranges are 5-42). The diagnostic work-up for growth cessation and delayed puberty were initiated, but all of the findings were normal: thyroid hormones, gonadotropins, and IGF-1 and anti-transglutaminase antibodies. Hand and wrist x-rays confirmed the development of ossification centers in relation to chronological age (the corresponding skeletal age was 13 years old). Because of the presence of polyuria, urine specimens were examined for spot and a 24-hour sample confirmed intense polyuria (6000 ml), high urinary calcium level (670 mg; v.n. 100-300) , with normal excretion of other electrolytes, and low urinary creatinine (660 mg/24 h; v.n. 800-2000). Creatinine clearance was reduced: 70 ml/min (v.n. >100 ml/min). We tested the plasma and urinary osmolarity which were normal; the ultrasonography of abdomen showed hyper echogenic liver and pyramidal calcification of kidneys with enhanced corticomidollary differentiation features suggestive of medullary nephrocalcinosis (Figure 1), and gallbladder lithiasis. Brain and pituitary gland magnetic resonance imaging did not reveal abnormality, neither in brain parenchyma nor Sella and parasellar regions. We checked calcitonin, parathyroid hormone, renin angiotensin aldosterone system and all results were found normal. Plasmatic, urinary amino acid levels and urinary acid organics did not reveal an abnormal pattern. In order to assess a possible cause for hyper transaminasemia lipid subset, alpha 1 antitrypsin, and liver kidney microsome tests were done. The complete infectivological panel did not reveal any abnormalities. The blood gas analysis showed a normal pH and bicarbonate level. The characteristics were compatible with an incomplete distal renal tubular acidosis that is the sign of WD. Finally the levels of serum concentration of copper and ceruloplasmin were 21 mcg/dl, v.n. 70-140 and 4 mg/dl v.n. 22-58 respectively which is another possible suggestion of WD. This suspicion was confirmed by elevated copper urinary excretion (111.30 mcg/24 hours’ urine; v.n. 0-50). Liver biopsy showed typical signs of WD when viewed on an electron microscope: mild lymphocytic infiltrate and eosinophilia granulocyte, no piecemeal necrosis, fatty liver degeneration (steatosis) to medium and small lipid droplets with moderate diffuse nuclear glycogenation, numerous Mallory bodies and lobular activity. Copper’s content was 270 μg/g dry weight of liver. Slit-lamp eye examination did not reveal the presence of typical Kayser-Fleischer ring, while a bone density scan revealed significant bone demineralization (Figure 2). Molecular tests confirmed, through direct DNA sequencing, the presence of a mutation in homozygosis N1270S (belong to exon 18). We started therapy with D-Penicillamine (20 mg/Kg/day) given in three doses, one hour prior meals, B-vitamin complex and a low copper diet. Moreover, we initiated therapy for osteoporosis with Alendronate (70 mg/weekly) and calcium and D vitamin complex. After two months a decrease in urinary copper and calcium excretion were revealed. The ultrasonography features of mild hepatitis responded to treatment and hypercalciuria was slightly decreased while nephrocalcinosis was unchanged.

## 3. Discussion

The prevalence of WD is reported 30 cases per million and its birth incidence rate is one per 30.000 to 40.000 (1, 2). Molecular genetic analysis reveals at least 300 distinct mutations of ATP7B gene that consist of 21 exons. The H1069Q mutation (exon 14), which is the most frequent mutation in the United States and central, eastern and northern Europe. This mutation has been associated with delayed onset of symptoms and less severe disruption of copper metabolism (3), although not all studies support this issue (4). In contrast, nonsense and frame shift mutations may correlate with earlier onset of symptoms and more severe alteration of copper metabolism (5). Data from non-European countries are poor (6). Although the primary pathogenetic defect of WD lies within the hepatobiliary system, the consequences of the relentless copper accumulation are played out on a multisystemic battlefield (7). The systemic involvement affects several organs such as brain and eyes. However, the recognition of the disease may be difficult because often clinical signs are absent or atypical. On this matter our patient showed an unusual early manifestation of nephrocalcinosis as the unique sign of WD.

Until today renal involvement in WD which is characterized by hypercalciuria was firstly reported by Litin in 1959 (8). Four out of his five patients had hypercalciuria and two of them had either nephrocalcinosis or nephrolithiasis. Azizi et al. in 1989 reported the case of another patient with hypercalciuria and nephrolitiasis and postulated a tubular defect in calcium absorption (9). In 2004 Kalra published the case of an Indian female with a history of three months of recurrent abdominal pain and acute episodes of vomiting, pain in left side related to bilateral renal stones without hydronephrosis (10). She even showed mild jaundice (started six months before) and hepatomegaly. The commonality of all these cases was an acute clinical picture of nephrolithiasis. The case described by Hoppe in 1993 regarded a male adolescent of 14 years old with a six-year history of hypercalciuria, nephrocalcinosis and nephrolitiasis, presented with a macrohaematuria (11). In this patient, renal involvement was documented two years before diagnosis of WD. Our case is different and peculiar from the previously described cases because the patient presented a very long history (10 years) of permanent hypercalciuria without any acute episodes of nephrolithiasis. The pathogenesis of hypercalciuria is not yet clear; it is defined as a urinary calcium excretion in eccess of 0.1 mmol/Kg per 24 h or 4 mg/kg per 24 h (12). It was postulated that this might be due to distal renal tubular dysfunction, secondary to an increased copper excretion that caused local damage in renal tubules. Moreover, another cause may be the increased mobilization of calcium from bones to neutralize systemic acidosis. It has been observed that patients affected by WD are able to maintain normal or near normal blood pH, like in our case, but are unable to maintain lower urine pH after ammonium chloride loading. This is suggestive of incomplete distal renal tubular acidosis (13). It is well known that the diagnosis of WD is based on the combination of clinical features, biochemical analysis and mutation detection. The diagnosis is easier when patients present a complete phenotype, but may be missed in asymptomatic individuals such as pediatric patient or patient with unusual symptoms, or the absence of typical neurological and hepatic manifestations. The frequency of various mutations, correlated with regional differences, shows the data from India is quite divergent, reflecting the ethnic diversity of this large country. The most common mutations in patients, mostly from North-West India, are located on exons 8, 12, 13, 15, 16 and 18 (6, 14). A p.N1270S mutation however, is distinguished by its common occurrence in a large number of groups, including Chinese, Korean, Japanese, Indian, Sicilian, Bulgarian, Egyptian, Brazilian, Italian, Turkish, and American. The frequency of this mutation among diverse ethnic groups suggests that this residue may represent a hot spot for mutation

All WD patients, even in the pre symptomatic stage, need lifelong drug therapy to prevent symptoms or disease progression (15). In general, pharmacological treatments include copper chelators and zinc salts. The ultimate aim of medical treatment is to reduce copper accumulation in the tissues and maintenance of low but adequate copper levels in the organism (16). Symptomatic patients have an extensive copper overload, and the primary purpose of pharmacological treatment is to obtain a negative copper balance. This is accomplished by chelation therapy with D-Penicillamine or Trientine until free serum copper and urinary copper excretion are within the normal range. In most cases this lasts six to 12 months or longer. Penicillamine therapy is more effective when a proximal renal tubular acidosis is present.

## 4. Conclusion

It must be emphasized that signs of renal involvement were present in our patient from the age of five and this clinical feature remained the only sign of WD for 10 years, without the appearance of any symptoms of neurological and hepatic involvement or progression of nephrocalcinosis to a nephrolitiasis. So we have concluded that hypercalciuria and nephrocalcinosis, as exclusive renal involvement, although rare, can represent an early and stable feature of disease. These findings should suggest investigating for the presence of WD, with the goal of starting specific treatment to prevent irreversible damages and long term complications.
